# Neuropsychiatric disorders among Syrian and Iraqi refugees in Jordan: a retrospective cohort study 2012–2013

**DOI:** 10.1186/s13031-015-0038-5

**Published:** 2015-03-29

**Authors:** Erica D McKenzie, Paul Spiegel, Adam Khalifa, Farrah J Mateen

**Affiliations:** Department of Neurology, Massachusetts General Hospital, 165 Cambridge Street, #627, 02114 Boston, MA USA; School of Medicine, Queen’s University, Kingston, ON Canada; United Nations High Commissioner for Refugees, Geneva, Switzerland; United Nations High Commissioner for Refugees, Damascus, Syrian Arab Republic; Harvard Medical School, Boston, MA USA

**Keywords:** Refugees, Health services, Syria, Iraq, Humanitarian emergencies, Psychiatry

## Abstract

**Background:**

The burden of neuropsychiatric disorders in refugees is likely high, but little has been reported on the neuropsychiatric disorders that affect Syrian and Iraqi refugees in a country of first asylum. This analysis aimed to study the cost and burden of neuropsychiatric disorders among refugees from Syria and Iraq requiring exceptional, United Nations-funded care in a country of first asylum.

**Methods:**

The United Nations High Commissioner for Refugees works with multi-disciplinary, in-country exceptional care committees to review refugees’ applications for emergency or exceptional medical care. Neuropsychiatric diagnoses among refugee applicants were identified through a retrospective review of applications to the Jordanian Exceptional Care Committee (2012–2013). Diagnoses were made using International Classification of Disease-10^th^ edition codes rendered by treating physicians.

**Results:**

Neuropsychiatric applications accounted for 11% (264/2526) of all Exceptional Care Committee applications, representing 223 refugees (40% female; median age 35 years; 57% Syrian, 36% Iraqi, 7% other countries of origin). Two-thirds of neuropsychiatric cases were for emergency care. The total amount requested for neuropsychiatric disorders was 925,674 USD. Syrian refugees were significantly more likely to request neurotrauma care than Iraqis (18/128 vs. 3/80, p = 0.03). The most expensive care per person was for brain tumor (7,905 USD), multiple sclerosis (7,502 USD), and nervous system trauma (6,466 USD), although stroke was the most frequent diagnosis. Schizophrenia was the most costly and frequent diagnosis among the psychiatric disorders (2,269 USD per person, 27,226 USD total).

**Conclusions:**

Neuropsychiatric disorders, including those traditionally considered outside the purview of refugee health, are an important burden to health among Iraqi and Syrian refugees. Possible interventions could include stroke risk factor reduction and targeted medication donations for multiple sclerosis, epilepsy, and schizophrenia.

**Electronic supplementary material:**

The online version of this article (doi:10.1186/s13031-015-0038-5) contains supplementary material, which is available to authorized users.

## Background

There are more than 50 million forcibly displaced persons worldwide [1]. At the close of 2013, there were 641,915 refugees in the Kingdom of Jordan, including 585,300 Syrian and 20,300 Iraqi refugees assisted by the United Nations High Commissioner of Refugees (UNHCR) [2]. There are limited reported data on neuropsychiatric disorders among Syrian and Iraqi refugees, and existing publications draw primarily on Iraqi populations [3,4]. Neuropsychiatric disorders in displaced populations remain under-studied in countries of first asylum, partly due to the difficulty in gathering clinically informative data during times of humanitarian crises. Knowledge of neuropsychiatric disorder patterns among refugees from middle-income countries, however, is relevant to displaced populations in many locations, particularly as the demographic transition occurs globally and refugees seek third-country resettlement [4].

Governments of countries of asylum together with UNHCR take responsibility for the health needs of refugees. Here, we analyze the clinical burden and demographic parameters of Syrian and Iraqi refugees requiring emergency and expensive neuropsychiatric care from the UNHCR in Jordan. We then analyze the direct cost of care for refugees with serious neuropsychiatric disorders.

## Methods

This study was reviewed by the Massachusetts General Hospital Institutional Review Board (protocol 2014P001102/MGH) and granted exemption due to use of an existing database.

### The Exceptional Care Committee

Primary and secondary care for refugees in Jordan are provided by government health centers and hospitals as well as through UNHCR partner clinics, using a cost-sharing model [5]. The UNHCR funds tertiary care for refugees of any age based on the cost and immediacy of care required via application to the Exceptional Care Committee (ECC). Applications are made on behalf of the refugee, and referral for tertiary care must be decided or approved by a physician. Cost is determined by the treatment facility, and care is rendered by the Jordanian system or UNHCR-partnered non-governmental organizations, including Caritas, Jordan Health Aid Society and International Medical Corps [5]. The UNHCR’s network includes 18 primary health care centers and 23 referral hospitals [5]. Standard operating procedures have been established to review requests for care for severe illnesses, either in anticipation of costly medical treatment or surgery or in response to an emergency medical intervention [6]. Services available in Jordan include neuroimaging, electrophysiological studies, acute stroke care, and neurosurgery. Guidelines for decision-making for exceptional care have been set forth by the UNHCR and include assessment of a patient’s clinical need, prognosis, and confirmation of registered refugee status [5,6]. The ECC states that it generally does not approve treatments that would not be available to the average Jordanian [5]. The ECC meets monthly in the Kingdom of Jordan and includes local specialist physicians as well as representatives from UNHCR and the World Health Organization.

### Data extraction and analysis

All applications to the ECC from January 1, 2012 to December 31, 2013 were retrospectively reviewed by two independent reviewers, including a neurologist (FJM), for extraction of neuropsychiatric diagnoses. Only Iraqi and Syrian refugees were included as separate groups since refugees from other countries of origin (e.g. Egyptian, Sudanese) were relatively small in number. Refugees were diagnosed by International Classification of Disease 10^th^ edition codes [7] by their treating physicians in the Kingdom of Jordan. Only diagnoses that were primarily neuropsychiatric in presentation and etiology were included. For example, a patient who presented for emergency care for bilateral feet ulcers and paraplegia with a history of spinal cord injury was excluded. We intended to include cases that would typically fall within the practice scope of a neurologist or psychiatrist. Psychiatric disorders here include serious mood and psychotic disorders. All other applications were considered “non-neuropsychiatric” presentations. Applications were coded as “surgery”, “emergency care,” or “medical treatment” by the UNCHR ECC.

The money applied for and approved by the UNHCR and its partners was reported in Jordanian dinars (JD). These values were converted to United States dollars (USD) using the mid-market exchange rate of 1 JD = 1.408 USD on January 1, 2013, the midpoint time of the study observation period [8]. Reported costs were rounded to the nearest USD.

All refugees were registered through UNHCR. A UNHCR registration number was available for the majority of applications, allowing for the identification of multiple applications by a single refugee. When an identification number was not reported (5%, n = 13), we assumed that each application was on behalf of a unique refugee. For patients with multiple applications, the age listed on the first application and the primary diagnosis were used in the analysis. Cost per applicant was the sum of all applications in the two-year period.

### Statistical analysis

Basic descriptive analyses were performed using mean, median, range, and interquartile range. Comparison of two means was performed using two-sample t-tests and proportions were compared using tests of two proportions. The level of significance was predetermined at p = 0.05. Calculations were performed using Stata version 11.2 (Austin, TX).

## Results

The ECC reviewed 264 applications made on behalf of 223 refugees (n = 89, 40% female). The median age was 35 years (1^st^ quartile 12 years, 3^rd^ quartile 55 years) for neurological (n = 187) and psychiatric (n = 36) care. This represented 11% (n = 264) of all ECC applications (n = 2,526) in two years. Most applications were for neurological (83%) versus psychiatric (17%) care. Most applications were for emergency care (67%; n = 176), followed by medical treatment (18%; n = 48), and surgical intervention (15%; n = 40) (see Additional file 1). Applicants (n = 223) originated from Syria (57%; n = 128), Iraq (36%; n = 80), and Sudan, Somalia, Egypt and Yemen collectively (7%; n = 15). Iraqi applicants were significantly older on average than Syrians (44 versus 33 years, p < 0.001).

### Financial burden of neuropsychiatric disorders

Funds requested to cover neuropsychiatric care totaled $925,674 (Table 1). Neuropsychiatric applications cost $3,506 per application, compared to non-neuropsychiatric applications, which averaged $3,449 per application. The average cost per applicant was higher for Iraqis compared to Syrians ($5,462 versus $3,550, p = 0.02). Syrians were more likely to be approved for funding than Iraqis (130/138 versus 82/102, p = 0.001). The average approved funding per applicant did not differ significantly between Iraqis and Syrians ($3,918 versus $2,818, p > 0.05). The ECC approved 80% (n = 210) of applications for the full amount, 5% (n = 12) of applications for less than the full amount, with an average of 62% of the requested funding granted, and 1% (n = 3) of applications for more than the full amount, with an average of 128% of the requested amount granted. The ECC denied 11% (n = 30) of applications, and 3% (n = 9) of applications were pending at the time of data collection.

**Table 1:** **Average amount of financial assistance requested for neuropsychiatric disorders, per application mean (median; range)**

	Age* (Years)	**Syrian (USD)**	**Iraqi (USD)**	All (USD)**
Neurological applications (n = 218)	0-5	2,411 (1,593; 563–12,674) (n = 36)	1,962 (1,538; 275–4,347) (n = 7)	2,338 (1,538; 275–12,674) (n = 43)
	6-19	3,930 (2,474; 829–20,762) (n = 21)	3,518 (3,137; 363–8,450) (n = 8)	3,695 (2,753; 363–20,762) (n = 31)
	20-50	6,845 (4,813; 563–26,371) (n = 25)	5,015 (3,591; 401–36,615) (n = 47)	5,517 (4,109; 401–36,615) (n = 78)
	>50	2,789 (1,525; 820–14,083) (n = 25)	3,847 (2,804; 444–17,936) (n = 35)	3,281 (1,732; 444–17,936) (n = 65)
	All Ages	3,805 (1,977; 563–26,371) (n = 108)	4,250 (3,156; 275–36,615) (n = 97)	3,942 (2,267; 275, 36,615) (n = 218)
Psychiatric applications (n = 46)^Φ^	6-19	820 (820; 763–878) (n = 2)	2,051 (1,482; 1,056-5,437) (n = 6)	1,484 (1,080; 213–5,437) (n = 10)
	20-50	1,287 (1,069; 754–3,390) (n = 22)	2,486 (2,253; 1,056-5,070) (n = 5)	1,509 (1,169; 754–5,070) (n = 27)
	>50	1,177 (1,081; 875–1,686) (n = 6)	-- (n = 0)	1,177 (1,081; 875–1,686) (n = 6)
	All Ages	1,232 (1,055; 754–3,390) (n = 33)	2,249 (1,638; 1,056-5,437) (n = 11)	1,441 (1106; 213–5,437) (n = 46)
All applications (n = 264)	All Ages	3,203 (1,619; 563–26,371) (n = 141)	4,046 (2,817; 275–36,615) (n = 108)	3,506 (1,921; 213–36,615) (n = 264)

Legend:

*Age missing for 1 neurological case and 3 psychiatric cases.

**Refugees from countries of origin other than Syria and Iraq are not shown.

^Φ^No applications for treatment of psychiatric disorders in refugees under 5 years of age were submitted in 2012 or 2013.

-- No applications in this category were reviewed by the exceptional care committee.

### Selected neurological diagnoses

There were 22 applications for traumatic injury of the nervous system: 17 cases of head trauma and 5 cases of spinal cord and peripheral nerve trauma. The median age was 24 years and 91% were male (Table 2). Traumatic injuries included both conflict-related events, such as bullet wounds, and unintentional injuries, such as falls in the elderly. Syrian refugees were significantly more likely to request trauma care than Iraqis (18/128 Syrians vs. 3/80 Iraqis, p = 0.03).

**Table 2:** **Frequency, cost and approvals of selected diagnoses among exceptional care committee applications for refugees**

	**Diagnosis**	**No. refugees (% Female)**	**No. of refugees with ≥ 2 applications**	**Total no. of applications**	**Total cost (Average per person) USD**	**% Applications approved**
Neurological	Stroke^Ω^	36 (47)	5	41	147,601 (4,100)	95
	Brain Tumor	23 (61)	6	29	181,815 (7,905)	73
	Traumatic Injury of the Nervous System	22 (9)	0	22	142,252 (6,466)	86
	Neurodevelopmental Abnormalities	17 (56)	2	19	44,688 (2,629)	94
	Disc Prolapse	16 (44)	0	16	65,462 (4,091)	50
	Meningitis	12 (42)	2	14	36,852 (3,071)	93
	Epilepsy	11 (27)	1	12	21,245 (1,931)	100
	Encephalopathy and LOC^Ψ^	8 (38)	0	8	21,444 (2,680)	100
	Cerebral Palsy	7 (57)	2	9	24,801 (3,543)	67
	Multiple Sclerosis	7 (57)	2	12	52,511 (7,502)	92
	Headache	5 (60)	0	5	9,412 (1,882)	100
Psychiatric	Schizophrenia	12 (17)	4	18	27,226 (2,269)	100
	Psychosis	8 (25)	0	8	10,596 (1,325)	100
	Bipolar Disorder	5 (60)	2	9	17,113 (3,423)	100

Legend:

^Ω^Stroke included three cases of transient ischemic attack.

^Ψ^LOC: Loss of consciousness.

Stroke was the most common neuropsychiatric diagnosis (n = 41 applications, 16% of neuropsychiatric applications; median age 64 years; 47% female). Nearly all applications were for emergency care (93%, n = 38) all of which were approved. Six refugees with stroke were reported to have later died.

Brain tumors accounted for 13% (n = 29) of neuropsychiatric applications, and was the most expensive neuropsychiatric diagnosis overall and per applicant. The ECC denied six applications for reasons of eligibility, cost, and/or prognosis. Of the 20 approved applications, 15% (n = 3) were approved for less than the requested amount, receiving on average 39% of requested funds.

### Psychiatric diagnoses

There were 46 applications for psychiatric care submitted on behalf of 36 refugees (median age 32 years; 28% female). Schizophrenia was the most common diagnosis (33%, n = 12), followed by psychosis not otherwise specified (22%, n = 8) and bipolar disorder (14%, n = 5). Most psychiatric applications were for emergency care (87%, n = 40). Requested funds for psychiatric care totaled $66,277, with each emergency application costing an average of $1,372. The ECC approved 98% (n = 45) of psychiatric applications.

### Pediatric applications

In refugees five years of age and younger (n = 38), the most common diagnoses were neurodevelopmental abnormalities (n = 15), cerebral palsy (n = 4), epilepsy (n = 4), and meningitis (n = 4). The ECC reviewed no psychiatric care applications for refugees under five. Among applicants aged six to nineteen years (n = 34), the most common diagnoses were traumatic injury (n = 6) and brain tumor (n = 4); seven adolescents, ages 12 to 17 years old sought care for psychiatric diagnoses. Requested funds for pediatric neuropsychiatric care were on average less than those requested for adult care ($3,246 versus $4,687, p = 0.02).

## Discussion

The protracted and violent nature of the conflicts in the Middle East has resulted in a large population of refugees facing long-term displacement, creating an unprecedented strain on host countries’ health systems [9]. Although neuropsychiatric disorders accounted for 11% of all ECC applications among refugees in Jordan, these disorders have received little attention. The ECC decisions distinguish diagnoses that are costly due to number of affected applicants compared to high individual care needs. Consistent with existing data on cancer care in the region, tumor care exceeded all other disorder category costs in this dataset [10]. Stroke was costly due to its higher frequency, while multiple sclerosis was expensive at an individual level.

Importantly, our results highlight opportunities for interventions for neuropsychiatric disorders among Syrian and Iraqi refugees, and may be relevant to refugees in future humanitarian crises. First, routine screening for hypertension as well as improving other risk factor management – including smoking cessation, diabetes screening, and improving physical activity levels – may lessen the burden of stroke [11]. These interventions have benefits to health in general beyond the nervous system and may be incorporated into routine health screening. Second, enhancing folic acid supplementation may help reduce the incidence of neurodevelopmental abnormalities, which were reported frequently to the ECC. Food scarcity and limited income to purchase food have been particularly concerning in the Syrian refugee crisis [12].

Third, investment by particular companies, including those in the pharmaceutical industry, may alleviate a patient group’s long-term burden of specific disorders and avert the need for future emergent care. An example of this is disease modifying drugs for multiple sclerosis. Whereas programming is well-established for poliomyelitis and other vaccine-preventable neurological disorders in which the rollout of vertical programs occur quickly [13], programs for stroke, epilepsy, multiple sclerosis, and traumatic brain injury are increasingly necessary. Both the host population and refugees may benefit in an integrated manner from the implementation of such non-communicable disorder treatment and prevention programs. Innovative models including public-private partnerships, crowd funding, and emphasis on preventative services have been proposed for the management of other non-communicable disorders in refugee populations [10].

Fourth, primary mental health care plays an essential role in reducing the number of psychiatric emergency applications among refugees. The UNHCR estimated that 1.6 million Syrians have been affected by violence [12], underscoring the high likelihood of covert psychiatric disorders and underreporting among the refugee populations in Jordan. Post-traumatic stress disorder and depression are nearly absent in the ECC files, despite the likelihood of their high prevalence in displaced populations. These disorders may also not incur the expensive care that the ECC was designed to support.

There are several limitations in our study. The ECC files lack several variables of interest, including patient outcomes and illness duration prior to application. Refugees not registered with the UNHCR were not eligible to have their applications reviewed by the ECC. Also, refugees who could afford to seek care through the private health care system may have done so without requesting ECC assistance. These numbers therefore underestimate the burden of disease in the source population. We were not able to measure the overall incidence and prevalence of neuropsychiatric disorders among Iraqi and Syrian refugees in Jordan or identify which disorders preceded asylum seeking.

This study also has several strengths. The ECC files are among the few available data sources on actual health provision in humanitarian crises. Diagnoses were made by physicians, using ICD-10 codes, and confirmed by diagnostic testing such as magnetic resonance imaging and biopsy. We were able to distinguish between visits and persons. By comparison, community-based surveys of non-communicable disorders in the region have relied on self-report, layperson interviewers, and usually lack diagnostic confirmation [11]. Refugee heath research in the region has primarily focused on Iraqis. Syrian and Iraqi refugees face different challenges, including differing exposure to violence and the duration of conflict and displacement. Health expenditure data, as presented here, have not been previously reported in studies of neuropsychiatric disorders in refugee populations [3,14]. Given the continued displacement within the region, and reports of host countries repealing healthcare aid [15], future priority-setting for refugee healthcare will require cost of care data and assessment of disease-specific treatment needs and outcomes.

## Conclusions

Taken together, the ECC decisions suggest that investment in relatively inexpensive primary and public health initiatives for neuropsychiatric disorders may reduce the demand for costly, exceptional, and emergency care amongst refugees. Such knowledge may improve the overall neuropsychiatric health of the refugee populations in Jordan and countries of future resettlement.

## Additional file

Additional file 1:
**Number of applications to the Exceptional Care Committee for refugees by age and category.**
