# Supplementary material for: Neuropsychiatric disorders among Syrian and Iraqi refugees in Jordan: a retrospective cohort study 2012–2013
Source: Confl Health. 2015 Mar 29;9:10. doi: 10.1186/s13031-015-0038-5 (PMC4406163; doi:10.1186/s13031-015-0038-5)
Supplement: Additional file 1: — Number of applications to the Exceptional Care Committee for refugees by age and category. [file 13031_2015_38_MOESM1_ESM.doc]

| **Appendix 1:** Number of applications to the Exceptional Care Committee for refugees by age and category | | | | | |
| --- | --- | --- | --- | --- | --- |
|  | Age** (Years) | Emergency  (# Applications) | Treatment  (# Applications) | Surgery  (# Applications) | Total  (# Applications) |
| Neurological | 0-5 | 34 | 5 | 4 | 43 |
|  | 6-19 | 20 | 8 | 3 | 31 |
|  | 20-50 | 34 | 25 | 19 | 78 |
|  | >50 | 47 | 4 | 14 | 65 |
|  | All ages | 136 | 42 | 40 | 218 |
| Psychiatric ^Φ^ | 6-19 | 7 | 3 | -- | 10 |
|  | 20-50 | 24 | 3 | -- | 27 |
|  | >50 | 6 | -- | -- | 6 |
|  | All ages | 40 | 6 | -- | 46 |
| All | All ages | 176 | 48 | -- | 264 |

Legend:

**Age missing for 1 neurological case and 3 psychiatric cases.

Φ No applications for treatment of psychiatric disorders in refugees under 5 years of age were submitted in 2012 or 2013.

-- No applications in this category were reviewed by the ECC.
